# Supplementary material for: “Live-Autoradiography” Technique Reveals Genetic Variation in the Rate of Fe Uptake by Barley Cultivars
Source: Plants (Basel). 2022 Mar 18;11(6):817. doi: 10.3390/plants11060817 (PMC8956111; doi:10.3390/plants11060817)
Supplement: Supplementary file 1 [file plants-11-00817-s001.zip › plants-1628817-supplementary.pdf]

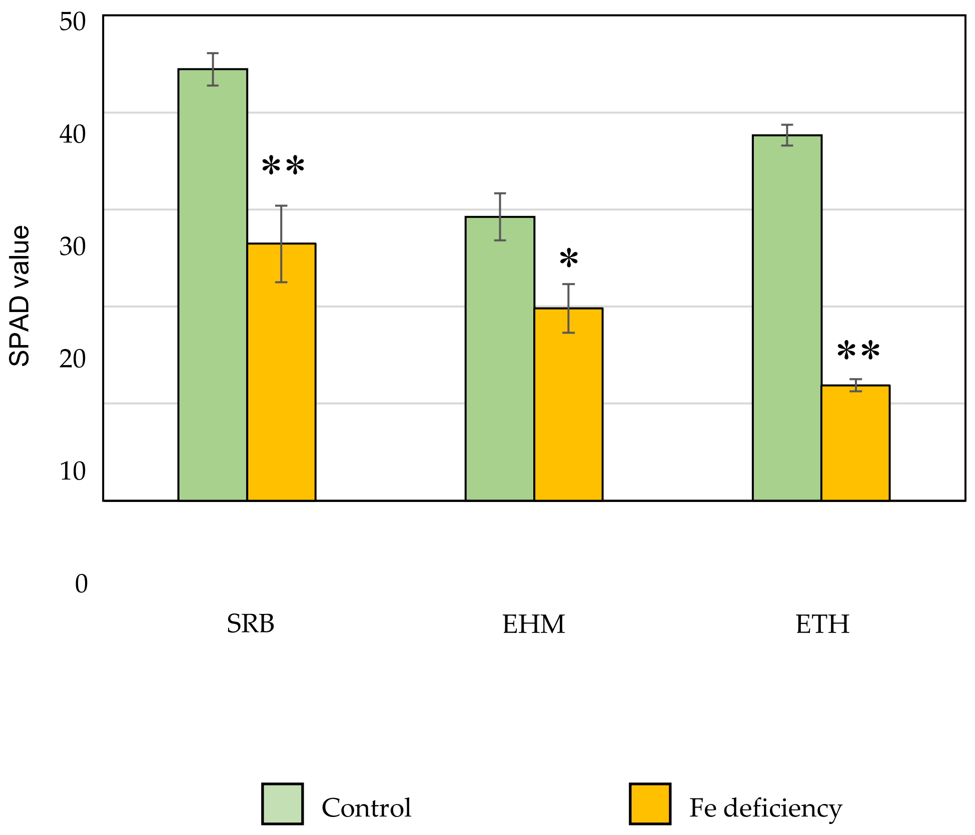

Figure S1. SPAD (chlorophyll meter) values of third leaves (L3 appeared in Fig. 1–3). SPAD-502 (Minolta, Osaka, Japan) was used. Values represent the means (SE) of four plants. ◎P < 0.05and ◎ ◎ P < 0.01 indicate significant differences (according to Student’s t-test) between control leaf andFe-deficient leaf.

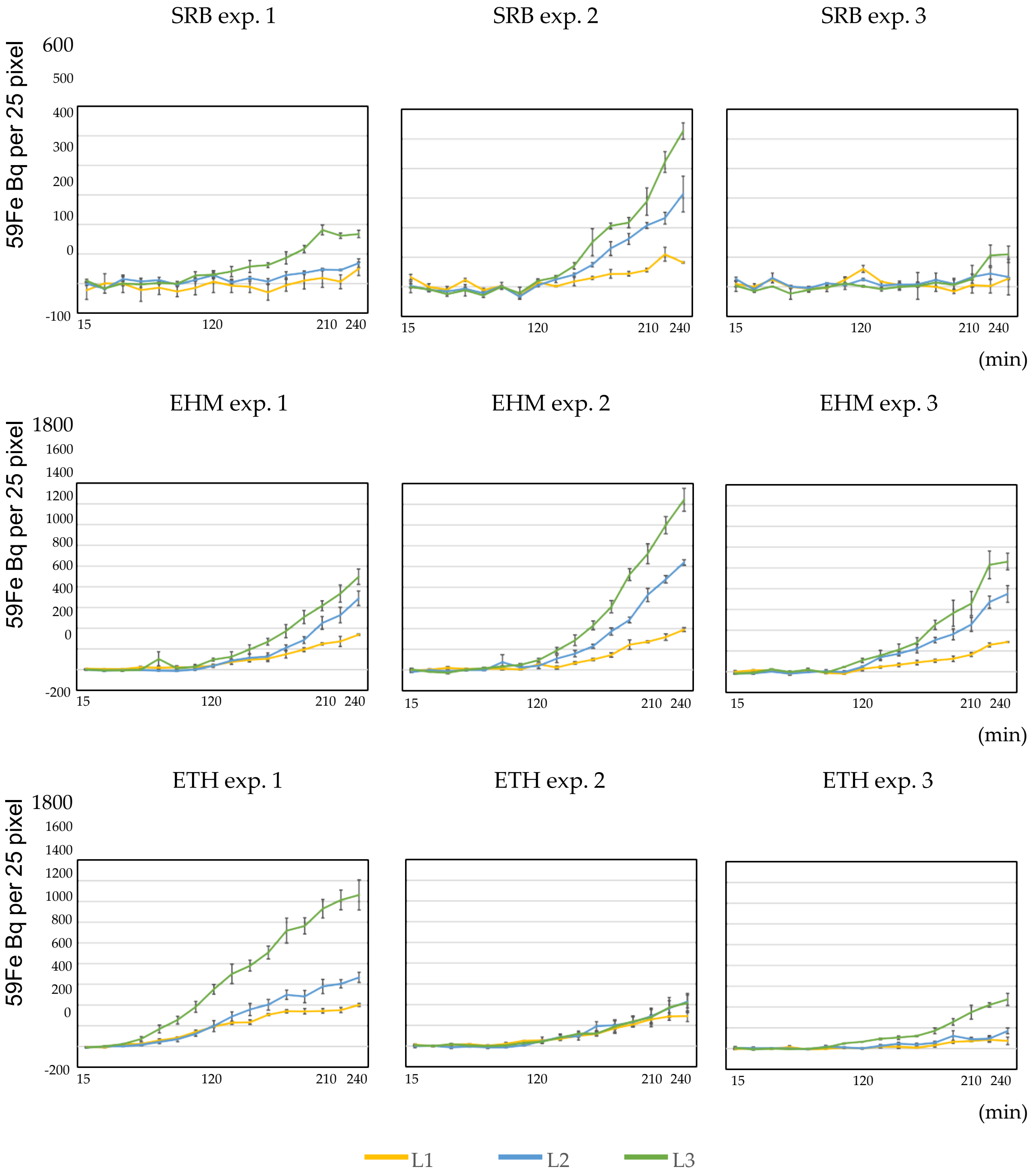

Figure S2. TAC of each Fe-deficient leaf.

Each experiment was executed with one plant. Each data point was calculated from threeROIs on one leaf, and S.E. was represented.

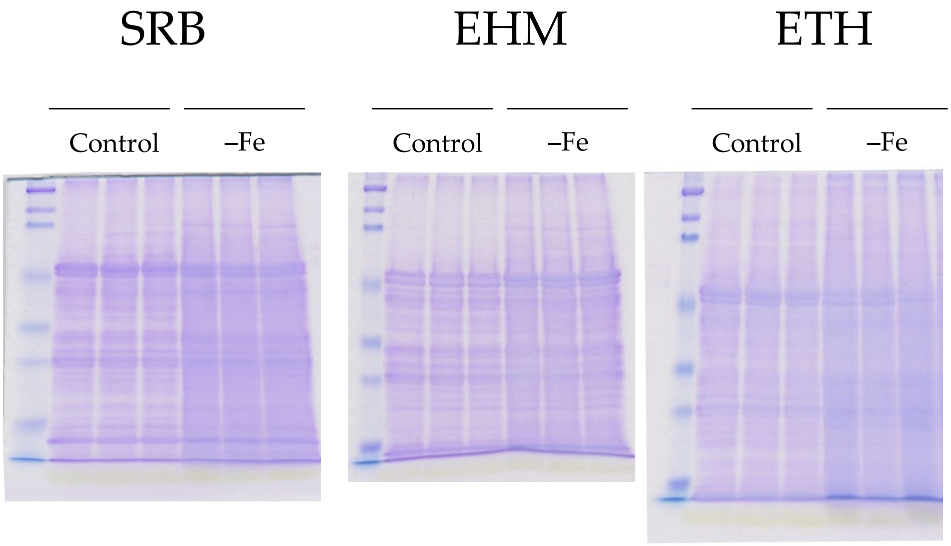

Figure S3. CBB staining of the same samples presented in Figure 4.Each lane was loaded 500μg of chlorophyll, thus larger amountsof proteins from Fe-deficient leaves were loaded than control leaves.

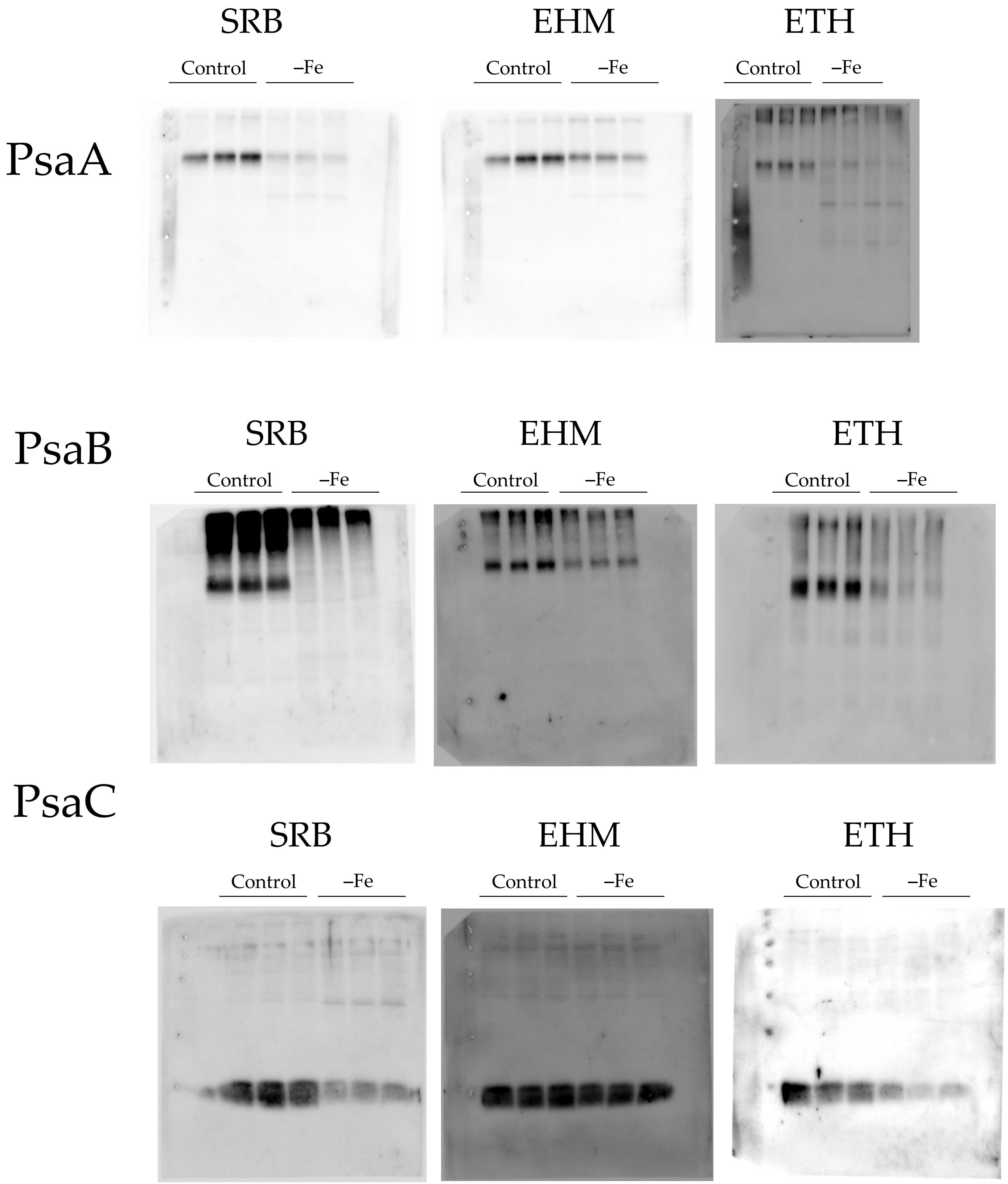
PsaA
SRB
Control
–Fe
EHM
Control
–Fe
ETH
Control
–Fe
PsaB
SRB
Control
–Fe
EHM
Control
–Fe
ETH
Control
–Fe
PsaC
SRB
Control
–Fe
EHM
Control
–Fe
ETH
Control
–Fe

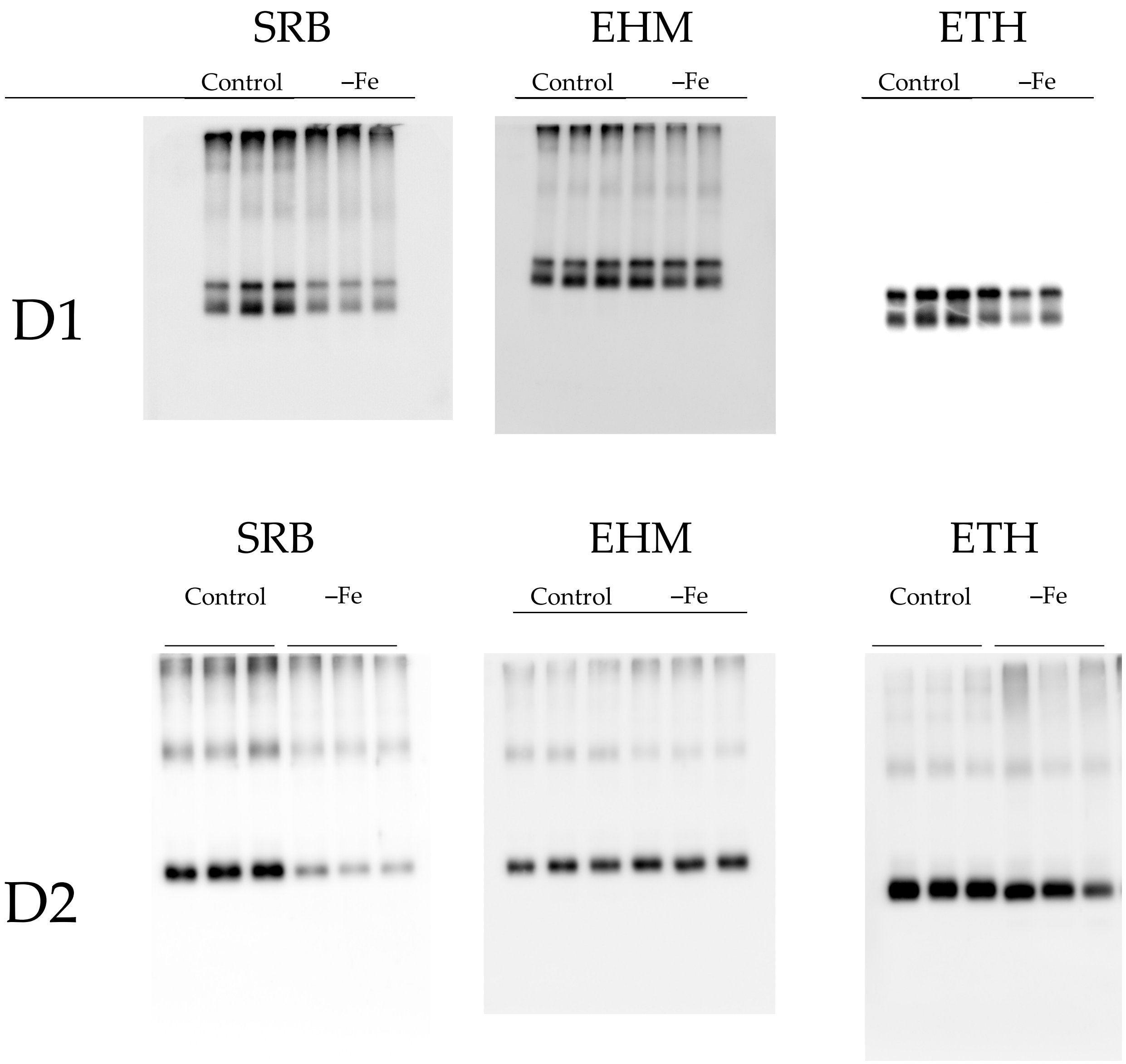

Figure S4. Original images of blots presented in Figure 5.

Table S1. Primers used for qPCR of SUF machinery genes.

Primer names	Forward sequence 5'-3'	Reverse sequence 5'-3'	Reference genes	amplified product (bp) for cDNA
HvSufB-Q	TCAGGTGGGTTGTACTAGTGG	GATCCCTCCAGCTTCAGGTT	HORVU3Hr1G087860.1, AK358935	213
HvGrxS14-Q	GTGCTGTTCATGAAGGGGAC	CCACTCTTGTATGCCTCGAG	HORVU5Hr1G122340.1, AK357754	230
EF1alphaQ-PCR	TGCTGCTGCAACAAGATGGA	TTGTACCAGTCAAGGTTGG	Z50789, AK249923, Z23130	188
Os+HvAct1Q-PCR	CAGCCACACTGTCCCCATCTA	GTGGTGAATGAGTAACCACGCTC	AK251023	129
